# Supplementary material for: Challenges and coping strategies faced by female scientists—A multicentric cross sectional study
Source: PLoS One. 2020 Sep 21;15(9):e0238635. doi: 10.1371/journal.pone.0238635 (PMC7505433; doi:10.1371/journal.pone.0238635)
Supplement: Attachment — Submitted filename: PONE-D-19-33911-Martin-report.doc [file pone.0238635.s002.doc]

**Challenges and coping strategies faced by female scientists —**

**a multicentric cross sectional study**

by Farah Naaz Fathima et al.

Submitted to *PLOS ONE,* 2019

**Reviewer’s report**

**Strengths of the paper**

The feminist movement has drawn attention to the disadvantages faced by women in a wide range of domains, of which science is an important one. Scientific careers seem to have been especially difficult for women, even though science might not seem to be so obviously a masculine domain. There have been many studies of the challenges faced by women in science.

This paper offers several valuable new angles to this issue. It analyses data from female scientists from around the world, especially in south and southeast Asia. This is a geographical area that warrants attention. The workplace factors raised provide valuable insight into the careers and concerns of female scientists. Especially useful are the findings concerning coping strategies, something given little attention in most studies.

After a brief setting of the stage, the study and its methods are clearly described. The findings are presented with adequate detail.

**Suggestions for improvement**

Given that the sample of female scientists is not random, but rather a convenience sample, it would be better to present the findings as indicative of major concerns in the cohort. Detailed figures and percentages are of less significance, and serve mainly to indicate the likely findings from a random sample. Furthermore, because the sample is worldwide, though with heavier representation from some areas, the findings cannot readily be said to apply to any specific area.

Similarly, the findings about coping strategies are only indicative. Nevertheless, they reveal self-understandings by female scientists.

To make the paper stronger, I think it would be useful for the authors to make their own assessments of how the coping strategies relate to the challenges. The most commonly cited coping strategies could be assessed in terms of whether they have the potential to address the challenges effectively. For example, one of the common challenges is workplace harassment. The authors could examine each coping strategy to determine whether it has a reasonable potential to address harassment. Hard work and dedication may not help a lot, and indeed harassment is often designed to undermine someone’s performance. Child care support seems unrelated to addressing harassment, whereas a supportive work environment would make a big difference. This little example then raises the question of whether a supportive work environment constitutes a strategy. Choosing a job where the environment is supportive could reduce the risk and impact of harassment, and trying to foster a more supportive environment might do the same.

The point here is not this example but the value of using the findings not as data giving accurate figures but as indicators of salient issues, specifically challenges and coping strategies. Assessing the main coping strategies in relation to the main challenges would make the study much more worthwhile. The authors might also use their collective experience and understanding to suggest strategies that would be more effective than those commonly reported.

In summary, the figures can be downplayed, the most common challenges and coping strategies highlighted, and connections between the challenges and strategies outlined.

**Lesser points**

• English expression should be polished.

******************

Scott Armstrong (Wharton School, University of Pennsylvania) for many years has studied and reviewed research on peer review. In order to encourage innovation, he recommends that referees do not make a recommendation about acceptance or rejection, but only comment on papers and how they might be improved, leaving decisions to editors. (See for example J. S. Armstrong, “Peer review for journals: evidence on quality control, fairness, and innovation,” *Science and Engineering Ethics,* Vol. 3, 1997, pp. 63-84.) I am persuaded by his arguments and hence make no formal recommendation about this paper.

As is my standard practice, I do not wish to be anonymous, and would be happy to correspond directly with the author.

Two short pieces of mine about refereeing appeared in the *Journal of Scholarly Publishing.*

“Writing a helpful referee’s report,” http://www.bmartin.cc/pubs/08jspwhrr.html

“Surviving referees’ reports,” http://www.bmartin.cc/pubs/08jspsrr.html

I’ve tried to follow my own advice for writing a helpful referee’s report; the ideas about surviving referees’ reports may be helpful to the author when making revisions.

Brian Martin

Emeritus Professor of Social Sciences

Humanities and Social Inquiry

University of Wollongong, NSW 2522

Australia

phone: +61 2 4221 3763

email: bmartin@uow.edu.au

http://www.bmartin.cc/

7 February 2020
