# Supplementary material for: Challenges and coping strategies faced by female scientists—A multicentric cross sectional study
Source: PLoS One. 2020 Sep 21;15(9):e0238635. doi: 10.1371/journal.pone.0238635 (PMC7505433; doi:10.1371/journal.pone.0238635)
Supplement: Attachment — Submitted filename: Response to Reviewers.docx [file pone.0238635.s003.docx]

**Response to Reviewers:**

**Reviewer 1:**

**Comment 1:**

This is a potentially important contribution on the topic that requires research and attention of the scientific community. The study is well designed and conducted. However, the quality of reporting can be strengthened further by using the STROBE checklist of items that should be included in reports of observational studies. I would like to encourage the authors to check their manuscript against the STROBE checklist and report the most relevant items in more detail. Additionally, the study would benefit from a more detailed elaboration of the survey questionnaire development. Finally, this recently published article might be of relevance: <https://journals.plos.org/plosone/article?id=10.1371/journal.pone.0225763>

**Response to comment 1:**

We thank the reviewer for the comment and the attached manuscript. We have used the STROBE checklist items for observational studies wherever applicable to our study.

**Reviewer 2:**

**General comment:**

See attached report. You will see that I'm recommending downplaying your quantitative results and instead using them as the basis for an assessment of how different coping strategies do or don't have a prospect of dealing with specific challenges.

**Response to the general comment:**

The authors agree with reviewer about the value of using the quantitative data for the assessment of coping strategies in dealing with the specific challenges and the authors considered this remark in the revised manuscript.

**Comment 1: Strengths of the paper**

The feminist movement has drawn attention to the disadvantages faced by women in a wide range of domains, of which science is an important one. Scientific careers seem to have been especially difficult for women, even though science might not seem to be so obviously a masculine domain. There have been many studies of the challenges faced by women in science.

This paper offers several valuable new angles to this issue. It analyses data from female scientists from around the world, especially in south and Southeast Asia. This geographical area warrants attention. The workplace factors raised provide valuable insight into the careers and concerns of female scientists. Especially useful are the findings concerning coping strategies, something given little attention in most studies.

After a brief setting of the stage, the study and its methods are clearly described. The findings are presented with adequate detail.

**Response to comment 1:**

The authors would like to thank referee for this comment. In fact as women scientists, we feel that women in science are still underrepresented, even in the developed world and we wanted to learn from other female scientists around the world about the challenges that they face and the coping strategies that they use to deal with.

**Comment 2: Suggestions for improvement:**

**Comment 2.1:**

Given that the sample of female scientists is not random, but rather a convenience sample, it would be better to present the findings as indicative of major concerns in the cohort. Detailed figures and percentages are of less significance, and serve mainly to indicate the likely findings from a random sample. Furthermore, because the sample is worldwide, though with heavier representation from some areas, the findings cannot readily be said to apply to any specific area. Similarly, the findings about coping strategies are only indicative. Nevertheless, they reveal self-understandings by female scientists.

To make the paper stronger, I think it would be useful for the authors to make their own assessments of how the coping strategies relate to the challenges. The most commonly cited coping strategies could be assessed in terms of whether they have the potential to address the challenges effectively. For example, one of the common challenges is workplace harassment. The authors could examine each coping strategy to determine whether it has a reasonable potential to address harassment. Hard work and dedication may not help a lot, and indeed harassment is often designed to undermine someone’s performance. Child care support seems unrelated to addressing harassment, whereas a supportive work environment would make a big difference.

This little example then raises the question of whether a supportive work environment constitutes a strategy. Choosing a job where the environment is supportive could reduce the risk and impact of harassment, and trying to foster a more supportive environment might do the same.

The point here is not this example but the value of using the findings not as data giving accurate figures but as indicators of salient issues, specifically challenges and coping strategies. Assessing the main coping strategies in relation to the main challenges would make the study much more worthwhile.

**Response to comment 2.1:**

As recommended, the authors used their assessment and experience in fieldwork on relating the coping strategies to the challenges. This point is discussed carefully for each coping strategy and updated studies references were used to emphasize the role supportive work environment in undermining the overwhelming challenges that female scientists face at familial and professional level.

**Comment 2.2:**

The authors might also use their collective experience and understanding to suggest strategies that would be more effective than those commonly reported.

In summary, the figures can be downplayed, the most common challenges and coping strategies highlighted, and connections between the challenges and strategies outlined.

**Response to comment 2.2:**

The authors have considered this point in the modified version and extensively discussed the different effective strategies that they have experienced to enrich the content of the manuscript.

**Comment 3 Lesser points:** English expression should be polished.

**Response to the comment 3 of lesser point:** The English language has been revised by all the authors and an English native speaker**.**
